# Advancing services for adult ADHD: the development of the ADHD Star as a framework for multidisciplinary interventions

**DOI:** 10.1186/s12913-016-1894-4

**Published:** 2016-11-08

**Authors:** Marios Adamou, Katharine Graham, Joy MacKeith, Sara Burns, Lisa-Marie Emerson

**Affiliations:** 1 Manygates Clinic, South West Yorkshire Partnership NHS Foundation Trust, Wakefield, Portobello Road, WF1 5PN UK; 2 Triangle Consulting Social Enterprise, The Dock Hub, Wilbury Villas, Hove, BN3 6AH UK; 3 Department of Psychology, University of Sheffield, Sheffield, UK; 4 University of Huddersfield, School of Human Health Sciences, Queensgate, HD13DH UK

**Keywords:** ADHD, Health services planning, Outcome monitoring

## Abstract

**Background:**

Attention Deficit Hyperactivity Disorder is prevalent in adulthood, resulting in serious impairment across multiple domains of living. Despite clinical guidance recommendations, the relative infancy of research on service provision for adults with ADHD, along with the evidence transfer gap, means that there is a lack of specific frameworks for service delivery. Igniting research and developing service delivery frameworks within adult ADHD is an essential step in the provision of effective services for adults with ADHD.

**Method:**

Following the methodology used in previous related research that utilises a Participatory Action Research approach, we gathered data from clinicians and service users on the domains of living in which they wish to create change, and the steps and end point of the change process. This data was utilised, alongside data gathered from previous research and policies, to develop the domains of assessment for the ADHD Star, and the scale on which change is assessed.

**Results:**

The resulting tool, the ADHD Star, consists of eight domains: understanding your ADHD, focus and attention, organising yourself, friends and social life, thinking and reacting, physical health, how you feel and meaningful use of time. Each domain is rated on a five-point scale, the ‘ladder of change’, ranging from ‘stuck’ to ‘choice’.

**Conclusions:**

The ADHD Star offers a guiding framework for the development of care pathways and subsequent service provision for adults with ADHD, based on multi-disciplinary, holistic and person-centred care.

## Background

Attention Deficit Hyperactivity Disorder (ADHD) is a neurodevelopmental condition characterised by inattention, hyperactivity and impulsivity, which present in at least two settings, interfering with functioning [1]. The mean worldwide prevalence of ADHD is between 5.29 and 7.1 % in children and adolescents (<18 years) [2, 3] and 4.4 % in adults [4]. It is now accepted that ADHD can persist into adulthood for the majority of individuals [5–7] and as a result, adults experience pervasive impairment across multiple domains including academic [8, 9], occupational [10], relational and self-concept [11] and is associated with psychiatric comorbidity [12], self-perceived stress [13] and poor health outcomes [14, 15]. Furthermore, adults with ADHD have increased mortality rates [16], linked to psychosocial adversity and unnatural causes, including accidents [17].

Research on adult ADHD has focused on symptomatic improvement with medication [18, 19] and non-pharmacological treatments [20]. However, the availability of these treatments is limited in many countries [21], due to limitations in mental health services, including awareness of the persistence of ADHD into adulthood and provision of sufficient services for these individuals. The scientific literature on health services for adult ADHD is similarly restricted, being derived mostly from children and adolescent populations, with only a small handful of studies conducted in the UK, describing small data sets [22–25]. However, the adverse consequences of ineffective service provision, particularly in the period of transition from paediatric to adult services, have been well and long articulated [24–27].

In addition to a dearth of research on service provision for adult ADHD, there are well-documented barriers to the application of any research evidence to the frontline of health care. The ‘evidence transfer gap’ has been linked to the size and complexity of the research, difficulties in developing evidence based clinical policy, ineffectual continuing education programmes and poor access to best evidence and guidelines [28]. For ADHD, there are additional disorder-specific barriers, including cognitive impairment [29], and stigmatisation by the public, peers and authorities [30–37]; furthermore, there is a distinct lack of clear therapeutic pathways for adult ADHD which health providers can adopt. A coherent framework for service provision for adults with ADHD is required in order to advance and improve existing services, and serve as a guide to new services. The current paper addresses this gap in service delivery research by describing the development of a recovery-based tool, the ADHD Star, and outlining how it can be used in services for adults with ADHD, for pathway planning, and the delivery and evaluation of the efficacy of treatments. In this paper, recovery is seen as a personal journey rather than a set outcome.

## Method

### Design and participants

The development process for the ADHD Star closely followed the established four-stage process for developing an Outcomes Star [38], and as described by Empirical Existential—Phenomenological Research [39]. In summary, the methodology draws on the core principles of Participatory Action Research, based on data collection, reflection and action [40]; clinical practitioners are involved in the research process from the initial design of the project, through data gathering and analysis, to final conclusions and actions arising out of the research [41]. The methodology was implemented over the course of three one-day workshops with Outcome Star experts, clinical practitioners and service users.

Experts from Triangle Consulting Social Enterprise facilitated the workshops with clinical practitioners in adult ADHD, between June 2013 and June 2014 in Yorkshire, UK. The facilitators have developed a number of Outcome Stars based on the same methodology since 2003 and are the definitive authorities internationally. Health and allied professionals from the Service for Adults with ADHD, South West Yorkshire Partnership NHS Foundation Trust participated in the workshops. This service was established in 2009 and has a catchment area of approximately 1.2 million people in West Yorkshire. The professionals involved were three Psychiatrists, two Clinical Psychologists, two Specialist Occupational Therapists, two Specialist Nurses, one Senior Clinical Pharmacist, one Principal Social Worker, one Senior Medical Liaison Advisor from a Pharmaceutical Company and two Senior Probation Service Officers. A diverse selection of service users and carers with links to this Service also participated in the data creation. This group had excellent knowledge of adult ADHD and involved the participation of representatives from two established service user and carer Charities.

### Procedure

#### Step 1. Problem and question formulation

During workshop 1, the facilitators delineated a focus of investigation to help formulate a hypothesis that the project would examine. The discussion was informed from previous research by the lead author [42].

Three key questions were derived:

i. What are the main areas in which services and service users seek to create change?
ii. What is the desired end point of the change process?
iii. What model of change describes the core steps that service users take on the journey towards the end point?

#### Step 2. Data-generating situation: protocol life-text

Focused exercises during workshops 1 and 2 generated data on participants’ subjective experiences of ADHD. A number of different techniques were used to draw out the experience and the implicit models that professional and service users and carers held about adult ADHD including:

i. Bringing to mind an individual who has undergone a substantial change and identifying the key areas of change.
ii. The use of metaphor and drawing to get a sense of the whole or essence of the change people undergo.
iii. Structured questioning exercises to draw out the change steps one by one in each outcome area; drawing out concrete information about the signs of change in great detail based on the experience of the professionals, service users and carers.

Further data was gathered from a review of the evidence base including key policy documents, National Guidelines, and relevant published research on adult ADHD.

#### Step 3. Data analysis: explication and interpretation

Following each workshop, the facilitators examined the transcripts and notes created by the participants and facilitators. A method of phenomenological sense-making described by Wertz [43] was followed; an iterative process of summarising, checking back against the data and then re-summarising revealed the structure, meaning configuration, principle of coherence, and the circumstances of occurrence and clustering. Repeating themes were identified, and underlying structures and meanings [e.g., core outcome areas or key stages of the journey] were drawn out. Reflection on wider knowledge gleaned through the development of other Outcome Stars informed the data analysis process.

#### Step 4. Presentation of results: formulation

The answers to the three questions outlined in Step 1 were presented back to the professionals in the form of a printed version of the ADHD Star for feedback and testing. Through an iterative process of sharing, listening, refining and sharing again, the outcome areas, the model of change and descriptions of the steps towards change in each outcome area were honed until they truly resonated with the professionals and service user and carers.

An initial draft of the ADHD Star was developed following workshop 1, and presented in workshop 2, at which point, further structured questioning generated feedback from all participants. An amended version of the ADHD Star was then piloted during a 6-month period. Fifteen adults accessing the Service for Adults with ADHD participated in the pilot through the completion of the ADHD Star with a health-care professional. This was a sample of convenience in which the Star was used as part of the Service day to day practice of care planning. They had combined type of ADHD, did not have any other comorbidity, 11 were male and 4 were female with an average age of 24 years old; professionals and service users recorded their experience through questionnaires. During the workshop 3, the results of the pilot were reviewed [including feedback and ADHD Star data] and the experience of participants was again shared. The feedback was mainly about reducing the steps of the ‘ladder of change’ from ten to five and comments about specific descriptors of change in the text. On the basis of this, further revisions were made to the ADHD Star and facilitators and participants approved the final version.

## Results and discussion

The aim of the current advancement was to develop a tool to serve as a framework to guide service provision for adults with ADHD. We *first* aimed to understand the aspirations and goals of service users and determine a meaningful and structured ‘journey of change’ across disorder-specific domains of need. Such an approach should define the desired outcomes of a person's life, based on the goals that they set for themselves. These goals should be individualised and consider: a decent place to live, employment and/or opportunity to contribute, education, friends and recreation outlets. Together, these outcomes will comprise the quality of one's life [44].

*Second*, we aimed to develop a tool that incorporated this knowledge in a way that was accessible to professionals and service users. Outcome Stars developed by Triangle Consulting Social Enterprise have already been successfully utilised in health services in the UK [45–47], therefore, a similar approach was adopted for developing a tool for adults with ADHD. In line with other Outcome Stars, dimensions relating to personal recovery were produced, with service user progress assessed along these dimensions. The ADHD Star consists of eight dimensions that relate to personal recovery (see Table 1). In collaboration with a health-care professional, service users rate each dimension on the ‘Ladder of Change’ (see Table 2) from ‘Being Stuck’ to ‘Self-Reliance’. Specific descriptors for each step of the Ladder of Change relating to adult ADHD were developed [see Table 3 for an example].

**Table 1:** The eight areas of the ADHD Star

1. Understanding your ADHD. This is about understanding how your ADHD affects you, and feeling you have some control over it. It covers getting diagnosed, making informed choices about treatment options, and being able to explain your behaviour to others and ask for what you need.
2. Focus and attention. This is about learning ways to help you pay attention to people and concentrating on tasks in a flexible way, so you can get things done.
3. Organising yourself. This is about the skills that you need to manage your life independently – managing time, sorting out your money, dealing with bills and paperwork, managing domestic tasks, not losing your possessions and coping with travel.
4. Friends and social life. This is about skills you need to have positive relationships with other people – family, friends, partners, colleagues, online friends and the wider community. It is about the quality of your relationships.
5. Thinking and reacting. This is about coping with strong feelings like anger and frustration. It is about managing negative impulses, like gambling, binge drinking, reckless driving or self-harm, thinking before you act, and not harming yourself or others, disrupting other people or damaging property.
6. Physical health. This is about how well you look after yourself – eating well, exercising, getting enough sleep, not misusing drugs, not smoking or drinking too much. It includes avoiding things that make managing your ADHD harder.
7. How you feel. This is about feeling positive, at ease and mostly ok about life. It is about accepting yourself, and being able to bounce back from life’s ups and downs, and cope with difficult emotions.
8. Meaningful use of time. This is about work, training or education – knowing what you want to do, building your skills and finding a meaningful occupation.

**Table 2:** Five steps on the ‘ladder of change’

1. Stuck. Service user may not be engaged or interested in change.
2. Getting help. Service user is starting to open up to help, but not yet taking imitative.
3. Trying things out. Service user is trying new things, but may give up easily if they do not seem to work.
4. Finding what works. Service users have made some achievements, and overcome barriers.
5. Choice and self-reliance. Service user is doing well and is on track with their recovery.

**Table 3:** Ladder of change descriptors from domain ‘Understanding your ADHD’

1. My life is chaotic and I don’t know why. No one is helping me.
2. My life is chaotic, but I have some help and have been given information about ADHD.
3. I’m trying to understand my ADHD and starting to try different options but this often doesn’t work.
4. I am learning what helps me cope with my ADHD, with some help.
5. I understand how my ADHD affects me and I mostly feel in control.

The ADHD Star addresses a gap in the current provision of services for adults with ADHD, offering a person-centred measure of progress in meaningful dimensions of change. Currently, instruments utilised with adults with ADHD in the UK healthcare system [48] focus on symptom measurement, highlighting deficit and impairment compared to the normal population. Many national guidelines recommend the assessment of deficit, and as such the focus of professionals and subsequent interventions is to remedy the deficits of the individual and on occasions their environment [49]. However, we know from previous work [42] that adults with ADHD have a different view on the types of interventions they would require, which are not limited to medicines. Rather, service users with adult ADHD desire a wider range of interventions, which the medical model alone cannot address. Therefore, a clinical approach based on achievement or ‘growth’ rather than ‘symptom reduction’ should be adopted for adults with ADHD. This view is consistent with the movement in the treatment of other psychiatric disorders [50, 51], and those that generate more person-centred care outcomes [52].

The ADHD Star offers a basis of care delivery for adults with ADHD, which has many advantages over current practice. First, it emphasises collaboration between service users and health-care professionals. Engagement with service users can be improved through the conversation generated by the tool. The outcome of this conversation is a shared care plan guided by adult ADHD specific domains that will resonate with the individual’s needs. A predicted consequence of this person-centred care is an improvement in adherence with proposed interventions and reduction in outpatient non-attendance rates. Second, the ADHD Star ensures that several domains of potential improvement will be considered as part of the assessment, thus opening the opportunity of multidisciplinary plans to be formulated and broader, holistic interventions applied. Third, the ‘ladder of change’ not only provides the opportunity to assess the service user’s current functioning, but also identifies the next step along that journey, thus ensuring robust goal-setting. Fourth, the ADHD Star enables different healthcare professionals to offer specialist input according to their skill and training across a specific domain, strengthening professional identity and specialisation. Fifth, when reviewed accordingly, the ADHD Star can be used as a tool to enable outcome-based commissioning.

There are some potential limitations to the implementation of the ADHD Star in healthcare services. Administration can take approximately two hours, which some services would deem too time-consuming. However, this time is spent in collaborative care-planning with the service-user, and will ensure meaningful goals are set and enhance commitment to agreed interventions. Therefore, we argue that ultimately, the time spent administering the ADHD Star will be saved elsewhere in failed interventions, and disengagement of service users. The ADHD Star was specifically designed to focus on meaningful outcomes for service users, and thus purposely omits ‘hard’ outcomes, i.e., symptom reduction. In isolation, the ADHD Star would neglect these outcome areas, which may be important to assess for monitoring the success of appropriate medical interventions as recommended by many authorities [53]. Thus, we recommend that the ADHD Star is used as part of a clear diagnostic and treatment pathway, such as the one outlined by the National Institute for Health and Clinical Excellence [54], to develop a care plan with clarity around multidisciplinary interventions and generate goals that are specific, measurable and realistic. If however some service users have co-occurrence with other disorders either mental illness [4] or neurodevelopmental disorders [55] that are the primary cause of impairment, a clinical decision needs to be made as to whether another tool should be used to chart the service user’s recovery journey.

## Conclusions

People with ADHD present in adulthood with impairments that have been developing for years, which are attributed to their symptom experience. As a result of this longevity, a framework is required to underpin a programme of evidence-based multidisciplinary interventions that have a clear direction and goals. We suggest that the ADHD Star offers such a framework and serves as a means to monitor outcomes for the purposes of service development. Future research should focus in investigating if the ADHD Star correlates with change on existing measures of ADHD-related impairments in quality of live or objective changes in an individual’s life such as changes in income, job status, housing, education and romantic relationship status.

## Abbreviations

ADHD: Attention deficit hyperactivity disorder
NICE: National Institute for Health and Care Excellence
